# Long non-coding RNA LSAMP-1 is down-regulated in non-small cell lung cancer and predicts a poor prognosis

**DOI:** 10.1186/s12935-022-02592-0

**Published:** 2022-05-06

**Authors:** Wei Gong, Yinyan Li, Jianfeng Xian, Lei Yang, Yuanyuan Wang, Xin Zhang, Yifeng Zhou, Xinhua Wang, Guibin Qiao, Cuiyi Chen, Soham Datta, Xincheng Gao, Jiachun Lu, Fuman Qiu

**Affiliations:** 1 The State Key Lab of Respiratory Disease, The First Affiliated Hospital, Guangzhou Medical University, 151 Yanjiangxi Road, Guangzhou, 510120 China; 2 The School of Public Health, The Institute for Chemical Carcinogenesis, Collaborative Innovation Center for Environmental Toxicity, Guangzhou Medical University, Xinzao, Panyu District, Guangzhou, 511436 China; 3 Department of Genetics, Medical College of Soochow University, 1 Shizi Road, Suzhou, 215123 China; 4 School of Public Health, Heping Development Zone, Gansu University of Chinese Medicine. No.1, Chinese Medicine Road, Lanzhou, 730101 Gansu Province China; 5 Department of Thoracic Surgery, Guangdong Provincial People’s Hospital, Guangdong Academy of Medical Sciences, Guangzhou, 510080 China; 6 Third People’s Hospital of Dongguan City, Dongguan, 523326 China; 7 Department of Urology, Minimally Invasive Surgery Center, The First Affiliated Hospital of Guangzhou Medical University, and Guangdong Key Laboratory of Urology, Guangzhou, Guangdong China

**Keywords:** *Lnc-LSAMP-1*, *LSAMP* gene, Non-small cell lung cancer (NSCLC), Biomarker, Prognosis, Chemosensitivity

## Abstract

**Background:**

Long noncoding RNAs (lncRNAs) are emerging as master regulators for gene expression and thus play a vital role in human tumorigenesis and progression. But the involvement of novel lncRNAs in non-small cell lung cancer (NSCLC) remains largely unelucidated.

**Methods:**

A total of 170 NSCLC and their adjacent non-tumor tissues were enrolled to detect the expression of *Lnc-LSAMP-1* by RT-qPCR. The effects of Lnc-LSAMP-1 on cell proliferation, migration, invasion and drug-sensitivity were determined by in vitro and in vivo experiments. The proteins that interact with Lnc-LSAMP-1were confirmed by RNA pull-down assay. RNA-sequencing were used to identify the potential targets of Lnc-LSAMP-1 in NSCLC.

**Results:**

We found that *Lnc-LSAMP-1* was significantly down-regulated in 170 cases of NSCLC tissues when compared to their adjacent non-cancerous tissues. Loss expression of *Lnc-LSAMP-1* was notably correlated with unfavorable prognosis of NSCLC patients. The ectopic expression of *Lnc-LSAMP-1* drastically inhibited lung cancer cell proliferation, viability, invasion and migration ability, arrested cell cycle and facilitated apoptosis. Chemotherapy sensitization experiments showed that over-expressed *Lnc-LSAMP-1* enhanced the inhibition of cell proliferation induced by TKI. Mechanistically, Lnc-LSAMP-1-LSAMP formed a complex which could protect the degradation of *LSAMP* gene, and thus exerted crucial roles in NSCLC progression and TKI targeted treatment.

**Conclusions:**

Consequently, our findings highlight the function and prognostic value of *Lnc-LSAMP-1* in NSCLC and provide potential novel therapeutic targets and prognostic biomarkers for patients with NSCLC.

**Supplementary Information:**

The online version contains supplementary material available at 10.1186/s12935-022-02592-0.

## Background

Lung cancer, one of the most common malignant tumors, has become the major causes of cancer-related deaths in the world [1, 2]. Non-small cell lung cancer (NSCLC) is responsible for approximately 85% of all lung cancer cases [3]. Although great progress has been made in the diagnosis and treatment of NSCLC in recent years, the overall 5-year survival rate for the disease in different regions and countries is between 4 and 17%, which is still at a low level [4–6]. Hence, the elucidation of a novel biomarker and the underlying molecular mechanisms associated with NSCLC progression is still imperative which may help accelerate the accurate diagnosis and targeted treatment for patients with NSCLC [7–10].

Long non-coding RNAs (lncRNAs) are a class of RNA molecules with more than 200 nucleotides in length that have no or limited protein-coding potential [11]. Accumulating evidence has demonstrated that lncRNAs participate in the regulation of diverse biological processes in cells such as cell proliferation, differentiation, apoptosis, migration and angiogenesis [12, 13]. LncRNAs have been identified as critical regulators in pathologic process at both transcriptional and post-transcriptional level [14–17]. Aberrant lncRNAs expression have identified to be involved in a various of cancers including lung cancer [18], breast cancer [19] and colorectal cancer [20]. Recent studies also have highlighted the lncRNAs expression profiling associated with cancer diagnosis, progression, prognosis, and response to drugs treatment [21–24]. Despite these findings, the functions and mechanisms of most aberrantly expressed lncRNAs in NSCLC development and progression remains incompletely interpretated.

Previous evidence indicates that a small region in 3q13.31 was frequently deleted and was a fragile area to malignancies [25, 26]. Genes identified within this genomic locus, have been strongly suggesting their tumor suppressor activity in cancers [26, 27]. Thereby, we performed a bioinformatics analysis to search candidate lncRNAs that locates in or nearby (with ± 400 kb distance) the 3q13.31 chromosome region using the UCSC genome database (http://genome.ucsc.edu/) and found several lncRNAs existed in this local region, which were also confirm with a public database for lncRNA sequence and annotation named LNCipedia (https://lncipedia.org/) [28]. By using the available web server GEPIA (Gene Expression Profiling Interactive Analysis) [29] (http://gepia.cancer-pku.cn/), we analyzed the relationships between these novel lncRNAs and NSCLC risk, as well as prognosis. We found that only the expression of *Lnc-LSAMP-1* was notably lower in NSCLC tissues, and was positively associated with the prognosis of patients with NSCLC. *Lnc-LSAMP-1* is located nearby a tumor suppressor gene termed limbic system-associated membrane protein (LSAMP) serving as an important membrane protein [30]. It is a pity that few studies were carried out the specific functions and regulatory mechanisms of *lnc-LSAMP-1* in lung cancer.

In the current study, we firstly investigate the expression *lnc-LSAMP-1* in the NSCLC tissues by RT-qPCR. The influences of *lnc-LSAMP-1* on cell proliferation, migration, apoptosis and targeted therapies were determined by CCK-8 assay, colony formation assay, transwell assay, flow cytometry and xenograft experiment in vitro and in vivo. The underlying mechanisms of *lnc-LSAMP-1 were* further explored by RNA-sequencing, RNA pull-down and RNA protection experiments. Intriguingly, we identified the lnc-LSAMP-1 was lowly expressed in NSCLC cancerous tissues compared with normal tissue, and its expression was positively associated with cancer stages and prognosis with great significance. Functional assays demonstrated that *Lnc-LSAMP-1* played a vital role in NSCLC growth and metastasis, and enhanced the inhibition of cell proliferation induced by TKI both in vitro and in vivo. Mechanistically, *Lnc-LSAMP-1* specifically binds to *LSAMP* to protect the degradation of *LSAMP* gene. Therefore, our findings establishing Lnc-LSAMP-1 /LSAMP regulatory axis may offer novel therapeutic targets for NSCLC patients.

## Materials and methods

### Patients and tissue samples

**Fig. 1:**
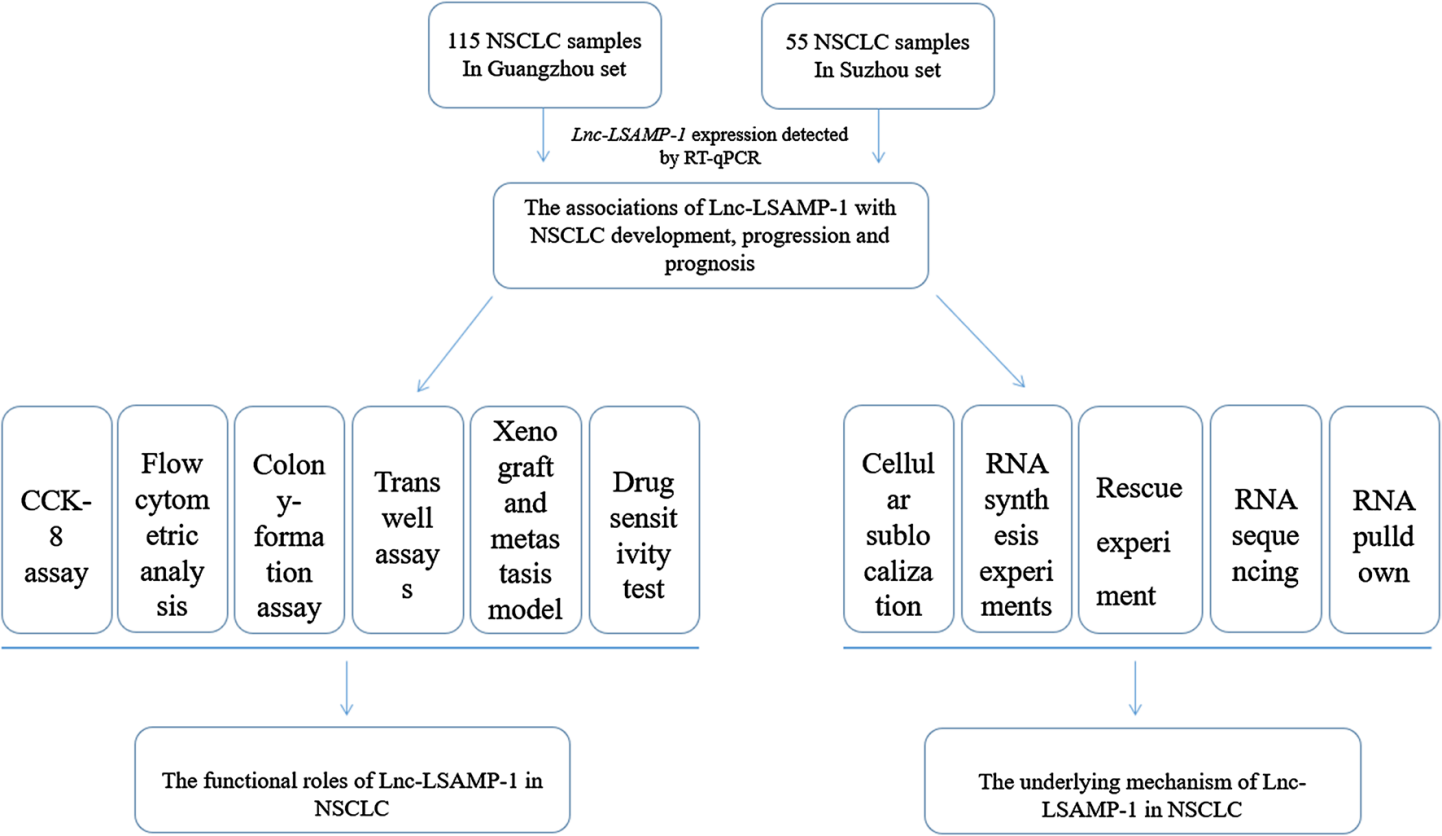
The experiment analysis flowchart in the current study

The study was fully approved by the Institutional Medical Ethics Committee of Guangzhou Medical University and Suzhou University. Written informed consent forms were obtained from all participants. A total of 170 NSCLC and their adjacent non-tumor tissue specimens were obtained from First Affiliated Hospital of Guangzhou Medical University, Affiliated Tumor Hospital of Guangzhou Medical University, and First Affiliated Hospital of Suzhou University. No patients received any radiotherapy or chemotherapy before surgery. All clinical information including age, gender, clinical stage, smoking history, infiltration degree, lymph node metastasis and distant metastasis, of these patients were collected recorded in a database. In addition, the fresh surgically removed tissues were immediately preserved in RNA later Solution (Thermo Fisher Scientific, US) and stored at −80 °C refrigerator. The design route of this study was summarized in Fig. 1.

### Cell culture

All the cell lines including A549, PC-9, NCI-H520, HCI-H460, L78, NCI-H292, GLC-82 human lung cancer cell lines, and HBE-pic, BEP-2D, BEAS-2B, 16HBE human Normal lung epithelial cell lines used in this study were purchased from Cell Bank of Type Culture Collection of the Chinese Academy of Science (Shanghai Institute of Cell Biology, China), and authenticated by STR typing. All cells were cultured in RPMI 1640 medium (Gibco, life technologies, California, USA) with 10% fetal bovine serum (FBS, Gibco, Thermo Fisher Scientific, Waltham, MA). Cells were placed in a CO_2_ incubator (SANYO Electric Co., Ltd., Japan) with constant 90% humidity and 5% CO_2_.

### Cell transfection

The full-length Lnc-LSAMP-1 cDNA sequence was synthesized by iGeneBio Co, Ltd, Company (Guangzhou, China) and cloned into the pEZ-Lv201 lentivirus expression vector (GeneCopoeia, China). The Lnc-LSAMP-1 vector or the empty vector were transfected into human 293 T cell to collect viral particles. The viral particles were then used to transfect lung cancer cell lines. After screening for 2 weeks using puromycin, the cells that stably expressed Lnc-LSAMP-1 were determined by RT-qPCR assay and cells that were infected with empty particles were used as control cells. Among the transfected lung cancer cell lines, A549 and PC-9 cells showed the best transfection efficiency, and they were chosen to perform subsequent functional experiments.

### Real-time quantitative reverse transcription PCR (RT-qPCR)

Total RNA from 170 paired NSCLC tissues and 11 cell lines were extracted using TRIzol reagent (Invitrogen, Carlsbad, California, USA). The total RNA was then transcribed to cDNA using commercial kits according to the manufacturer’s instructions (TaKaRa, Japan). RT-qPCR reaction (DBI, Germany) was performed in the Applied Biosystems 7900 Fast Real-Time PCR system (Applied Biosystems, CA, USA). β-actin was used as the endogenous control. The primers were synthesized by Sangon Biotech Ltd (Shanghai, China). The primer sequences used for RT-qPCR were presented in Additional file 4: Table S1. The 2^−ΔΔCT^ was used to demonstrate the expression levels of *Lnc-LSAMP-1* and *LSAMP*. All the experiments were conducted in triplicate.

### Western blotting

Western blotting was performed as previously described [31]. Briefly, total protein lysates from lentivirus-transfected-A549 and PC-9 cells were separated by 10% sodium dodecyl sulfate–polyacrylamide gel electrophoresis (SDS-PAGE), transferred to polyvinylidene difluoride (PVDF) membranes, then the membranes were washed and blocked. Primary antibodies of LSAMP purchased from Abcam (Cambridge, MA, USA) were applied to membranes, followed by horseradish-peroxidase-conjugated secondary antibodies according to the manufacturer's instructions.

### Cell proliferation assay

Cell proliferation assay was performed with Cell Counting Kit-8 (CCK-8, Corning Corporation, USA). Logarithmic phase cells were seeded into 96-well plates and were cultured for 12, 24, 36 and 48 h, respectively [32]. The absorbance of each well was read on a Thermo Scientific™ VarioskanTM LUX plate reader (Thermo Instruments, USA) (detection wavelength was 450 nm and the reference wavelength was 600 nm). A dynamic cell monitoring was also performed using the Incucyte Zoom Live-Cell Imaging System (IncuCyte ZOOM, Essen BioScience Co., Ltd., USA). Cell confluence was calculated by phase-contrast images according to the manufacturer’s protocols.

### Flow cytometric analysis of cell cycle and apoptosis

The flow cytometry analysis was used to identify whether *Lnc-LSAMP-1* influences cell cycle and apoptosis. For cell cycle analysis, the stable transfected cells were trypsinized (without EDTA), washed with PBS, and fixed with 70% ethanol at 4 °C for 12 h and resuspended in staining buffer containing 450 µl propidium iodide (PI) and 50 µl RNaseA in the dark for 30 min at room temperature. Then the flow cytometric assays (FACScan; BD Biosciences, Shanghai, China) were performed according to the manufacturer's instructions.

For cell apoptosis analysis, Annexin V/7-AAD apoptosis kit (MultiSciences, HangZhou, China) was used to stain for early and late apoptotic cells according to the manufacturers' protocols. Cells were washed twice in PBS and re-suspended in 1 × Binding Buffer to achieve a cell concentration of 1.0 × 10^6^ cells/ml. Subsequently, 10 ul of 7-AAD reagent and 5 ul of Annexin V reagent were added into cell suspension and stored for 30 min at room temperature in dark place. Apoptotic cells were examined and quantified using flow cytometry (Becton Dickinson, Lincoln Park, NJ, USA).

### Colony-formation assay

The cells were trypsinized and seeded into 6-well plates at a density of 200 cells/well. After 10 days of culture, cell clones that had formed from individual cells were directly observed by eye and then the colonies were washed with PBS, fixed 5% paraformaldehyde and stained with 0.1% crystal violet solution, followed by air-drying. The stained colonies were photographed, and counted using ImageJ 8.0 software (National Institutes of Health).

### Transwell assays

For the Transwell migration assay, the cells were trypsinized, adjusted to a concentration of 4 × 10^5^/ml, and seeded into the upper chamber with a non-coated membrane with 200 µl per well (24-well insert, pore size 8 μm; Corning, NY, USA). Lower chambers were supplemented with 10% fetal bovine serum (600 μL). After being incubated for 24 h with 5% CO_2_ at 37 °C, the upper surface of the membrane was removed with a cotton tip, while the cells on the lower surface were fixed using formaldehyde and then stained with 0.1% crystal violet for 30 min. Ten fields were randomly selected under a 100 × microscope and the number of cells that migrated to the lower layer was counted. For the invasion assay, as it is identical to the migration experiment procedure with coating with matrigel chambers (BD Biosciences, San Jose, CA, USA) were carried out according to manufacturer’s instructions. Briefly, transfected Lnc-LSAMP-1 cells (2 × 10^4^ cells/200 µl per well) were collected, resuspended in medium without serum, and then shifted to the hydrated matrigel chambers. The bottom chambers were incubated overnight in 600 μL culture medium with 10% FBS. The cells on the upper surface were scraped, whereas the invasive cells on the lower surface were fixed, colored and counted.

### Tumorigenicity and metastasis assay in nude mice

0.2 mL of cells suspension that contained 1 × 10^7^ cells was subcutaneously injected into the necks of 5 four-week-old female nude mice per group (Beijing Huarongkang Biotechnology Co. Ltd). Tumor growth was examined every 3 days for at least 1 month by measuring the length and width of the tumor mass. The experimental procedures for tumor metastasis model were similar to tumor growth model. 5 × 10^7^ cells were injected into caudal vein of nude mice (five mice per group). All mice were kept until death due to the neoplastic progression or until the end of the experiment (6 weeks). After 6 weeks, the mice were euthanized. Mice were injected intraperitoneally with barbiturate in a dose of 150 µg/g (total injection volume, 0.4 mL). After approximately 30 min, the mice were then sacrificed and the lungs were collected to evaluate the number of pulmonary metastatic lesions. Hematoxylin and eosin (H&E) staining was performed for tissue morphology evaluation following relevant protocols and strict operating procedures after soaking and fixing with 4% paraformaldehyde. All experimental procedures were approved by the Animal Ethics Committee of Guangzhou Medical University.

### Hematoxylin–eosin (HE) staining

The tumor masses from the nude mice were harvested and immersed in 4% paraformaldehyde for 4 h, and transferred to 70% ethanol. After that, the tissues were placed in processing cassettes, dehydrated through a serial alcohol gradient, and embedded in paraffin wax block, and then cut into 3-µm-thick sections that were baked at 45 °C for 5 h. Sections were then stained with HE (artificial hematoxylin and eosin) according to the following steps: 30 min of xylene dewaxing, treated with ethanol at different concentrations (100%, 90%, 70%), hydrated in distilled water, stained with hematoxylin (15 min), differentiated in hydrochloric acid ethanol and ammonia water, dehydrated with ethanol at 70% and 90% concentrations (10 min), stained with eosin ethanol (3 min), dehydrated with ethanol and cleared with xylene, and tumor tissue sections were then observed under a microscope.

### Subcellular fractionation

Nuclear and cytoplasmic fractions were isolated from A549 and PC-9 cells using the nuclear/cytoplasmic isolation kit (Biovision, San Francisco, CA) according the manufacturer's protocols. The RNAs from cytoplasmic and nuclear were then extracted using TRIzol reagent (Invitrogen, Carlsbad, California, USA). At last, qRT-PCR was performed to assess the relative expressions of β-actin (cytoplasm control), U6 (nucleus control), and *Lnc-LSAMP-1* in in each sample.

### Actinomycin D inhibits RNA synthesis experiments

Over-expressed *Lnc-LSAMP-1* or empty-control cells were seeded into 24-well plates at 5 × 10^4^ per well. After 24 h, the cells were treated with actinomycin D at a concentration of 2 mg/L. After 30 min, 1 h, 2 h, 3 h and 4 h, the expression levels of *LSAMP* gene were detected by qRT-PCR.

### Drug sensitivity test of lnc-LSAMP-1 on cisplatin and TKI (Tyrosine kinase inhibitors)

The IC50 of A549 and PC-9 cells was first screened using cisplatin concentration gradient (10 ug/ml, 5 ug/ml, 2.5 ug/ml, 1.25 ug/ml, 0.625 ug/ml, 0.3125 ug/ml), and finally determined as 5 ug/ml for A549 and 2.5 ug/ml for PC-9. In the preliminary experiments, several TKI drugs were also used to evaluate the effect of lnc-LSAMP-1 on therapeutic sensitivity, and found that only Nilotinib treatment were observed to have fulfilling inhibition rate changes induced by lnc-LSAMP-1. Nilotinib was then subsequently used as mean plasma drug concentrations such as Nilotinib = 3.6 μmol/L. High expression of *lnc-LSAMP-1* and control cells were treated with cisplatin and Nilotinib. The cells were inoculated into a 96-well plate, and the number of cells per well was 5.0 × 10^3^. Nilotinib was added after adhering to the wall and growing to about 10%, cisplatin was added up to 30–40%, and the 96-well plate was placed in a dynamic cell observer for cell proliferation detection (IncuCyte ZOOM. Essen BioScience Co. Ltd. USA). Proliferation inhibition rate = (experimental group (dosing)−experimental group)/(control group (dosing)−control group) × 100%.

### Rescue experiment

The rescue experiment was performed to validate that *Lnc-LSAMP-1* regulated lung cancer cell biological behaviors and enhanced the cell cytotoxicity induced by TKI treatment through targeting *LSAMP*. *LSAMP* inhibitor and blank inhibitor were transfected into A549 and PC-9 cells. The silencing efficiency of each siRNA targeting *LSAMP* was measured by qRT-PCR assay. The IncuCyte ZOOM long time live cell image monitoring system (Essen BioScience Co., Ltd., USA) was used to detect cell proliferations to evaluate the inhibition effect induce by *LSAMP*.

### RNA pulldown assay

For RNA pulldown assays, Biotin-labeled *Lnc-LSAMP-1* and its antisense were transcribed in vitro with the Biotin RNA Labeling Mix and T7 RNA polymerase (Roche, Basel, Switzerland), and then treated with RNase-free DNase I (Roche) and 0.2 M EDTA to stop the reaction. Biotinylated RNAs were mixed with streptavidin agarose beads (Life Technologies, Gaithersburg, MD) at 4 °C overnight. Total cell lysates were freshly prepared and added to each binding reaction with Protease/Phosphatase Inhibitor Cocktail and RNase inhibitor, incubated with rotation for 1 h at 4 °C. The RNA–protein binding mixture was separated using SDS-PAGE and the eluted proteins were detected by western blot.

### RNA sequencing

The total RNA from over-expressed Lnc-LSAMP-1 and empty-control A549 cells were extracted with TRIZOL reagent. The RNA concentrations and purities were measured using NanoDrop 2000 spectrophotometer (Thermo Electron Corporation, USA). The transcriptome sequencing was prepared using HISAT2 for Illumina® according to the manufacturer protocol (Guangzhou, Promege Biotechnology Co., Ltd). Raw reads were aligned to the human genome GRCh38 by Bowtie2. Differentially expressed genes between the two cell groups were identified using the expected number of Fragments Per Kilobase of transcript sequence per Millions base pairs sequenced (FPKM) method. The |log2(FoldChange)|> 1 and *P* value < 0.05 was as the threshold to judge the significance of gene expression differences.

### Bioinformatics analysis

Differentially expressed mRNAs were further analyzed with Gene Ontology (GO) enrichment analysis and the Kyoto Encyclopedia of Genes and Genomes (KEGG) pathway to investigate the functions and underlying mechanisms of *Lnc-LSAMP-1* in NSCLC progression.

### Statistical analysis

All statistical data were analyzed using the SPSS 16.0 software (SPSS, Chicago, USA). The differences of gene expression between lung cancer tissues and adjacent lung normal tissues were evaluated using paired-*t* test. The *χ*^2^ test was applied to analyze the distribution of gene expression between the demographics and clinical characteristics subgroup. Correlation between *Lnc-LSAMP-1* level and *LSAMP* expression was tested with the Pearson correlation analysis. The Log-rank test and Cox regression analyses were used to assess the effect of *Lnc-LSAMP-1* expression on lung cancer survival. Additionally, *P* < 0.05 was considered statistically significant (*P < 0.05; **P < 0.01; ***P < 0.001).

## Results

### Lnc-LSAMP-1 was dramatically down-regulated in NSCLC patients

As presented in Additional file 6: File S1, there were 22 lncRNAs existing nearby the 3q13.31 region. Among them, only *Lnc-LSAMP-1* was significantly associated with NSCLC risk and prognosis. So, we chose this lncRNA as candidate gene for further studies.

**Fig. 2:**
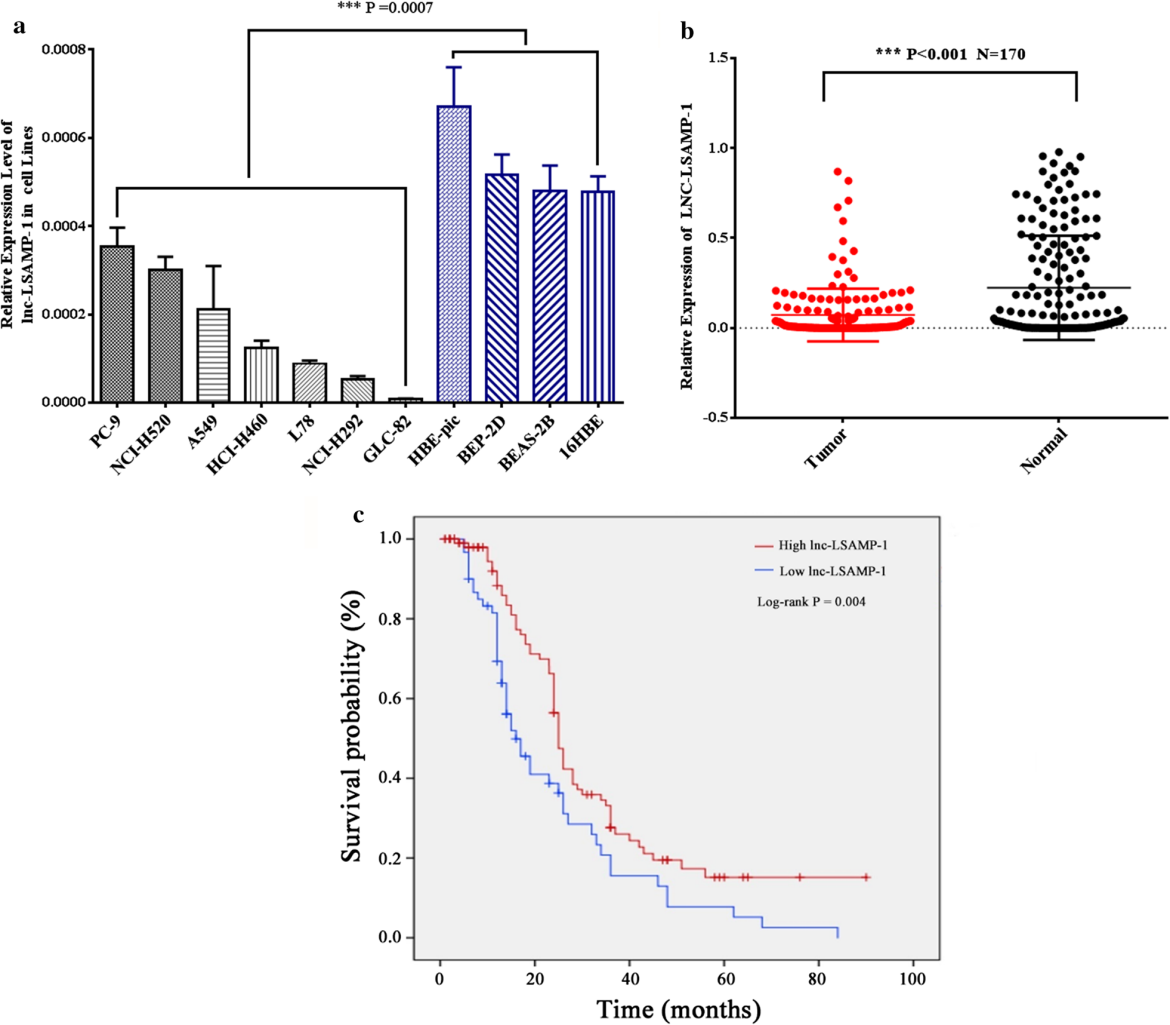
The expression patterns of *Lnc-LSAMP-1* in NSCLC cell lines and tisssues and its associations with NSCLC survival outcome. **a** The expression of *Lnc-LSAMP-1* in lung cancer and normal cell lines **b** *Lnc-LSAMP-1* expression levels in 170 pair-matched tumor tissues and adjacent normal tissues measured by RT-qPCR. **c** High expression level of *lnc-LSAMP-1* indicated better prognosis in NSCLC patients by Kaplan–Meier analysis (P = 0.004)

As shown in Additional file 1: Fig. S1a, analyzed by the online TCGA data platform GEPIA, the expression of Lnc-LSAMP-1 was observably lower in NSCLC tissues compared with normal tissues (*P* < 0.01). This positive finding was verified in lung cancer cell lines two independent NSCLC tissues. The demographics and clinical characteristics of studied patients were listed in Additional file 5: Table S2. Compared to human immortalized lung normal cell lines, the expression levels of *Lnc-LSAMP-1* were obviously down-regulated in lung cancer cells (*P* = 0.0007, Fig. 2a). Homoplastically, *Lnc-LSAMP-1* expression in the NSCLC tissues was proved to be significantly lower in comparison with the adjacent tissues in a total of 170 NSCLC cases (*P* < 0.001, Fig. 2b).

### Lnc-LSAMP-1 expression is correlated with clinical stage and N status

We further analyzed the relationship between *Lnc-LSAMP-1* expression and NSCLC clinicopathological characteristics. The group status of *Lnc-LSAMP-1* was classified based on the ratio of *Lnc-LSAMP-1* expression in lung cancer tissues versus that in adjacent normal lung tissues. If the ratio > 1, it was assigned to the high expression group, whereas if ratio < 1, it was identified to the low expression group. We found that the expression of *Lnc-LSAMP-1* was negatively correlated with clinical stage (*P* = 0.006) and N status (*P* = 0.009), and these findings were in accordance using TCGA database analysis as they presented that *Lnc-LSAMP-1* expression was prominently relevant with T status (*P* = 0.0349), N status (*P* = 0.0012), and stage (*P* = 0.0049) (Additional file 1: Fig. S1b, c and d). Nevertheless, no any notable association was observed between *Lnc-LSAMP-1* expression and other clinical features including age, gender, family tumor history, smoking and histological classification (all *P* > 0.05), as shown in Table 1.

**Table 1:** The associations between Lnc-LSAMP-1 expression and clinical characteristics of NSCLC in two datasets

Characteristic	Southern samples N (%)			Eastern samples N (%)			Total N (%)		
	Low expression	High expression	*P* value	Low expression	High expression	*P* value	Low expression	High expression	*P* value
Age									
< 60	40 (69.0)	18 (31.0)	0.155	19 (63.3)	11 (36.7)	0.311	59 (67.0)	29 (33.0)	0.508
≥ 60	32 (56.1)	25 (43.9)		19 (76.0)	6 (24.0)		51 (62.2)	31 (37.8)	
Gender									
Female	21 (67.7)	10 (32.3)	0.489	10 (62.5)	6 (37.5)	0.722	31 (66.0)	16 (34.0)	0.833
Male	51 (60.7)	33 (39.3)		28 (71.8)	11 (28.2)		79 (64.2)	44 (35.8)	
Family tumor history									
No	62 (60.8)	40 (39.2)	0.407	36 (70.6)	15 (29.4)	0.767	98 (64.1)	55 (35.9)	0.593
Yes	10 (76.9)	3 (23.1)		2 (50.0)	2 (50.0)		12 (70.6)	5 (29.4)	
Smoking									
No	25 (64.1)	14 (35.9)	0.813	11 (73.3)	4 (26.7)	0.929	36 (66.7)	18 (33.3)	0.715
Yes	47 (61.8)	29 (38.2)		27 (67.5)	13 (32.5)		74 (63.8)	42 (36.2)	
Stage (TNM)									
I + II	35 (74.5)	12 (25.5)	**0.029**	16 (84.2)	3 (15.8)	0.078	51 (77.3)	15 (22.7)	**0.006**
III + IV	37 (54.4)	31 (45.6)		22 (61.1)	14 (38.9)		59 (56.7)	45 (43.3)	
T status									
1 + 2	47 (66.2)	24 (33.8)	0.312	13 (54.2)	11 (45.8)	**0.035**	60 (63.2)	35 (36.8)	0.635
3 + 4	25 (56.8)	19 (43.2)		25 (80.6)	6 (19.4)		50 (66.7)	25 (33.3)	
N status									
0	34 (75.6)	11 (24.4)	**0.021**	22 (75.9)	7 (24.1)	0.251	56 (75.7)	18 (24.3)	**0.009**
1 + 2 + 3	38 (54.3)	32 (45.7)		16 (61.5)	10 (38.5)		54 (56.3)	42 (43.8)	
M status									
0	54 (65.1)	29 (34.9)	0.382	23 (62.2)	14 (37.8)	0.111	77 (64.2)	43 (35.8)	0.820
1	18 (56.3)	14 (43.7)		15 (83.3)	3 (16.7)		33 (66.0)	17 (34.0)	
Histological classification									
Adenocarcinoma	39 (65.0)	21 (35.0)	0.632	15 (68.2)	7 (31.8)	1.000	54 (65.9)	28 (34.1)	0.752
Squamous carcinoma	16 (55.2)	13 (44.8)		13 (68.4)	6 (31.6)		29 (60.4)	19 (39.6)	
Other types^a^	17 (65.4)	9 (34.6)		10 (71.4)	4 (28.6)		27 (67.5)	13 (32.5)	

Bold indicates statistically significant

^a^Large cell carcinoma, small cell carcinoma and hybrid or undifferentiated carcinoma

### Down-regulation of Lnc-LSAMP-1 predicts a poor prognosis in NSCLC

The potential prognostic value of *Lnc-LSAMP-*1 on NSCLC survival outcome was further evaluated. As suggested by the TCGA database analysis, the patients with reduced *Lnc-LSAMP-1* expression had a lower overall survival time than those cases with high-expressed *Lnc-LSAMP-1*(shown in Additional file 1: Fig. S1e). In addition, the undesirable role of *Lnc-LSAMP-1* on lung cancer prognosis was confirmed in our dataset and as shown in Fig. 1c, the NSCLC patients with low *Lnc-LSAMP-1* expression had a shorter survival time and worse prognosis (*P* = 0.004), supporting the argument for its utility as a biomarker for NSCLC prognosis.

### Lnc-LSAMP-1 suppresses cell proliferation

**Fig. 3:**
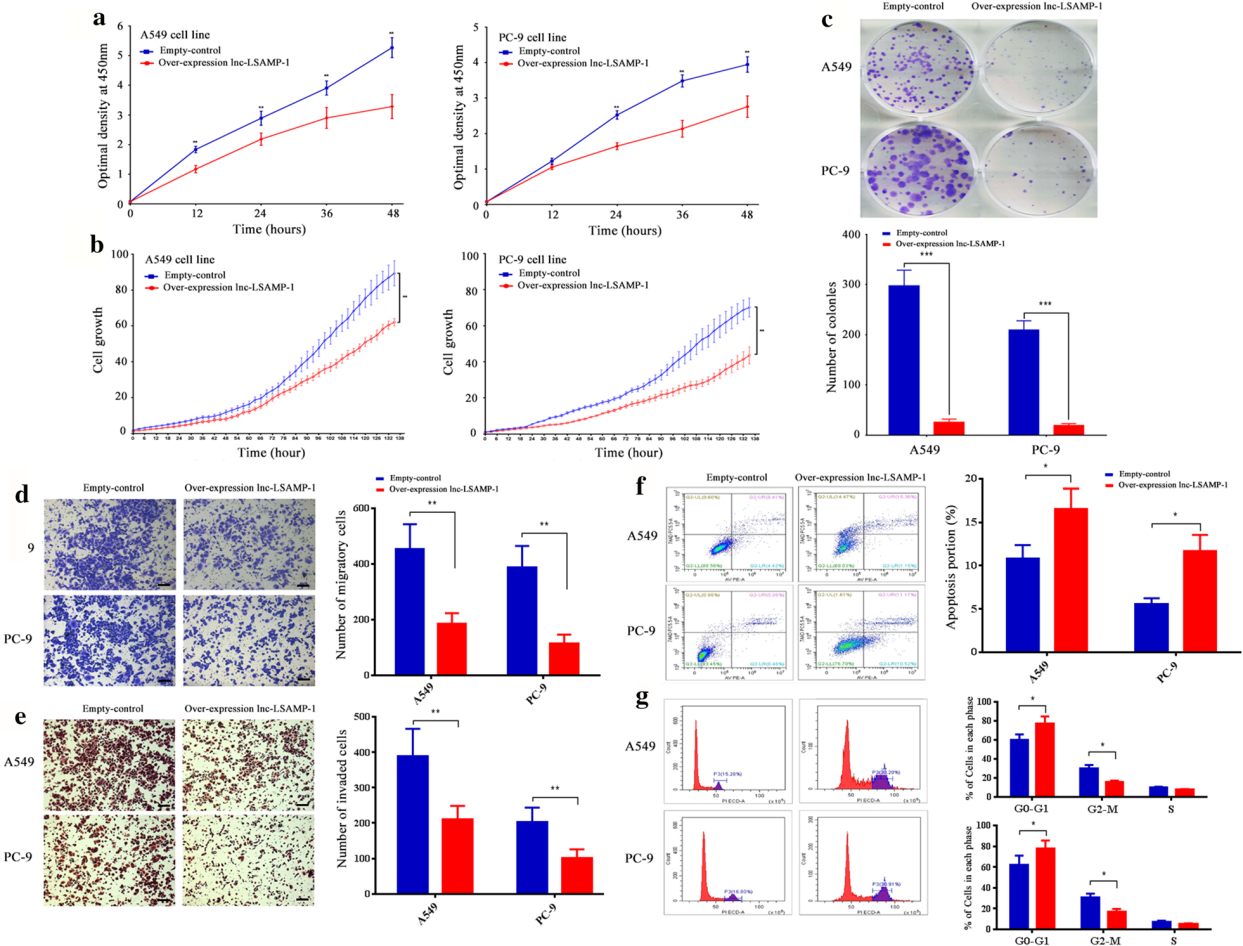
The effects of *Lnc-LSAMP-1* on NSCLC biological phenotypes in vitro. **a** CCK8 assay was performed to determine the cell proliferation. **b** Cell proliferation assay in Incucyte zoom (Essen BioScience Co., Ltd., USA). **c** clone formation was performed to determine the cell proliferation. **d**, **e** Migration and invasion capacities determined by Transwell assays. **f** The flow cytometry was conducted to determine the cell apoptosis of A549 and PC-9 cells. **g** The flow cytometry was conducted to determine the cell cycle of A549 and PC-9 cells. The results from three independent experiments, showed as mean ± s.d, and scale bar: 100 μm; Significance was defined as p < 0.05 (*p < 0.05; **p < 0.01; ***p < 0.001)

To determine the effect of *Lnc-LSAMP-1* on lung cancer cell viability and proliferation in vitro, CCK-8 and colony formation assay showed that the over-expressed *Lnc-LSAMP-1* could obviously suppress the cell proliferation in a time-dependent manner (*P* < 0.05, Fig. 3a). The same results were further observed in the dynamic cell viewer (*P* < 0.05, Fig. 3b). The plate colony formation assay also indicated that the cells with over-expressed Lnc-LSAMP-1 displayed fewer numbers of cell clones when compared to those cells with empty control (*P* < 0.05, Fig. 3c).

### Lnc-LSAMP-1 affects cell migratory and invasive abilities

In order to investigate the biological function of *Lnc-LSAMP-1* in NSCLC cell invasiveness, the Transwell assays was then executed. As shown in Fig. 3d, the over-expressed *Lnc-LSAMP-1* resulted in attenuated cell migratory abilities while compared to those cells transfected with empty vector both in A549 and PC-9 cell lines (all *P* < 0.01). Similarly, the results from the invasion assay demonstrated that the invasive ability in upregulated *Lnc-LSAMP-1* cells was significantly suppressed compared with control cells (*P* < 0.01, Fig. 3e).

### Lnc-LSAMP-1 affects cell cycle and induces apoptosis

As shown by flow cytometry analysis in Fig. 3f, over-expressed *Lnc-LSAMP-1* significantly enhanced the apoptosis rate both in A549 and PC-9 cells with respect to those control cells (all *P* < 0.05). Accordingly, cell cycle analysis showed that a significant increase in the percentage of G0/G1 phase (*P* < 0.05) and a corresponding marked decrease in the M phase (*P* < 0.05) was induced in the cells with high *Lnc-LSAMP-1* expression when compared to empty control cells (Fig. 3g).

### *Lnc-LSAMP-1 inhibits tumor growth *in vivo

**Fig. 4:**
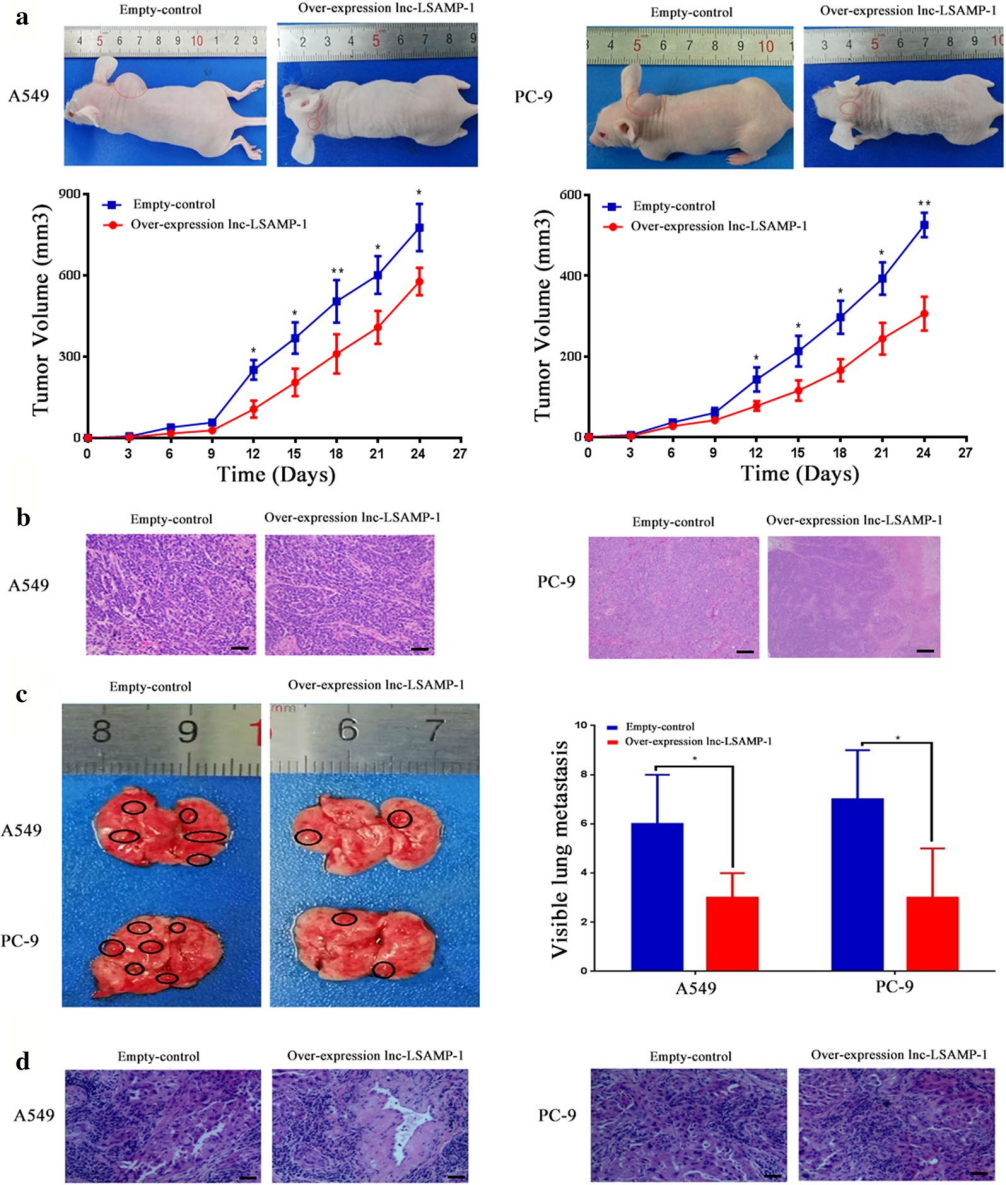
Overexpression of *Lnc-LSAMP-1* inhibited NSCLC tumorigenesis and metastasis in vivo. **a** Nude mice inoculated subcutaneously with A549 and PC-9 cells. Tumor growth rate and tumor volume are compared between *Lnc-LSAMP-1* overexpression group and control group. **b** A subcutaneous tumor-forming HE section of nude mice in xenograft. **c** The lung suspected metastases in nude mice with A549 and PC-9 cells injected via tail vein. The black circle indicated the suspected cancerous lesions in the naked eyes of sacrificed nude mice. **d** A HE slice of suspicious lung nodules in nude mice injected with tail vein injection. Scale bar: 100 μm. Significance was defined as p < 0.05 (*p < 0.05; **p < 0.01; ***p < 0.001)

The subcutaneous xenograft model and metastasis model was applied to validate the biological function of *Lnc-LSAMP-1* in vivo. As shown in Fig. 4a, b, c and d, consistent with the results in vitro, the nude mice injected with over-expressed *Lnc-LSAMP-1* cells revealed a lower tumor volume than those nude mice injected with control cells (all *P* < 0.05). Accordingly, in comparison to those in the control group, the severity and number of metastatic lesions in mice inoculated with over-expressing *Lnc-LSAMP-1* were significantly depressed (all *P* < 0.05). Our findings indicated that *Lnc-LSAMP-1* could suppress NSCLC cell proliferation and metastasis both in vitro and in vivo.

### The potential mechanism of Lnc-LSAMP-1 inhibited NSCLC growth and metastasis

**Fig. 5:**
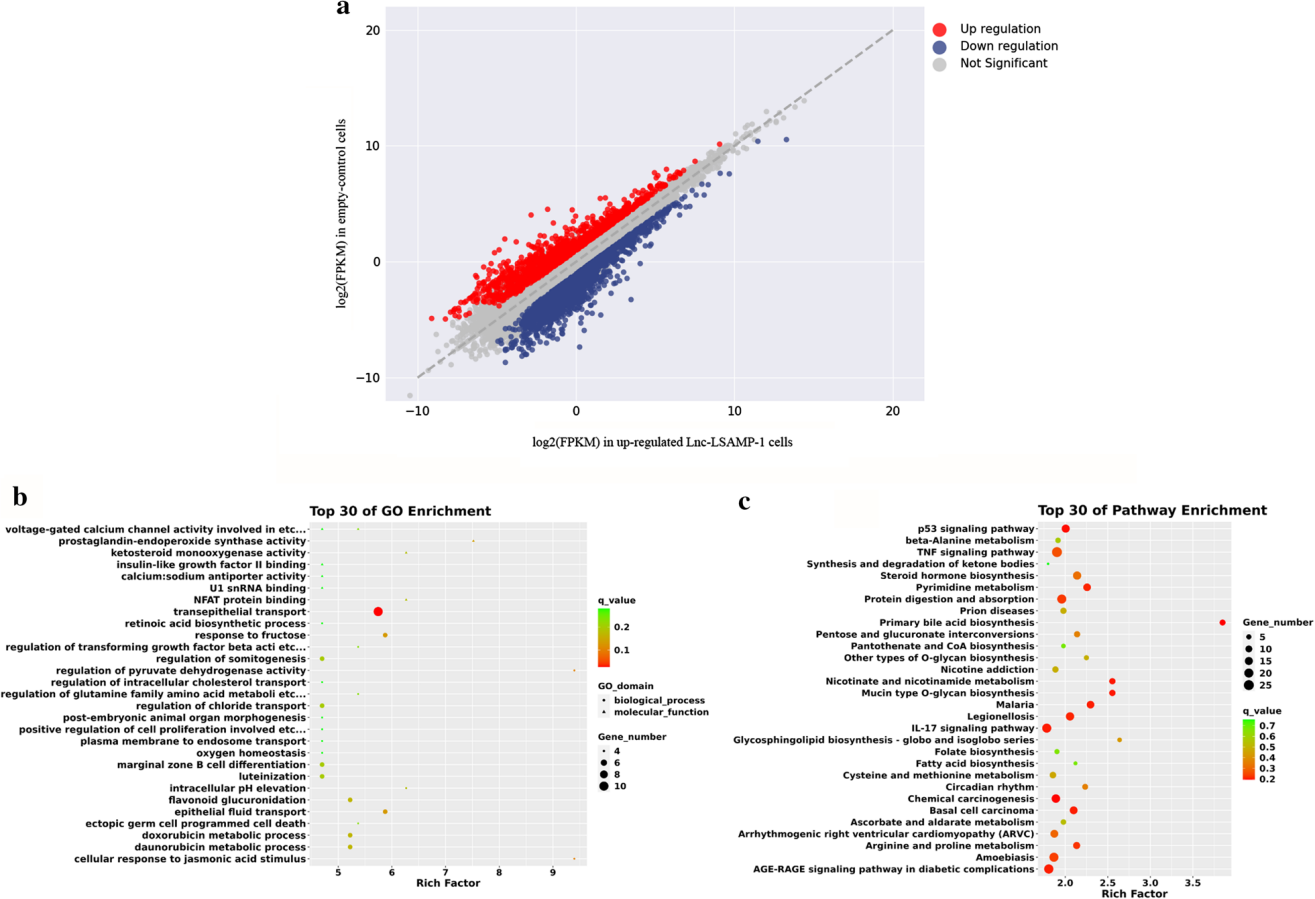
The RNA-sequencing results and bioinformatics analysis. **a** The differentially expressed genes induced by *Lnc-LSAMP-1.* **b** GO analysis and the top 30 results presented. **c** KEGG pathway analysis and the top 30 results presented

To further investigate the mechanism by which *Lnc-LSAMP-1* suppressed malignant phenotype of NSCLC cells, we conducted the transcriptome sequencing between the over-expressed *Lnc-LSAMP-1* and control cells. The fold change of gene expression was calculated and genes with |log2(FoldChange)|> 1 and P value < 0.05 were considered as differentially expressed. As shown in Fig. 5a, a total of 3692 genes were identified to be differentially expressed induced by *Lnc-LSAMP-1.* Among them, 1552 genes were significantly up-regulated, while 2140 genes were memorably down- regulated. GO analysis revealed that dysregulated mRNAs were significantly enriched in several biological processes, such as transepithelial transport, metabolic process and epithelial fluid transport. KEGG pathway analysis showed that these DEGs were mainly enriched in p53 signaling pathway, TNF signaling pathway and Chemical carcinogenesis (Fig. 5b and c), and most of them were cancer-related.

### Lnc-LSAMP-1 interacted with LSAMP and maintained its expression

**Fig. 6:**
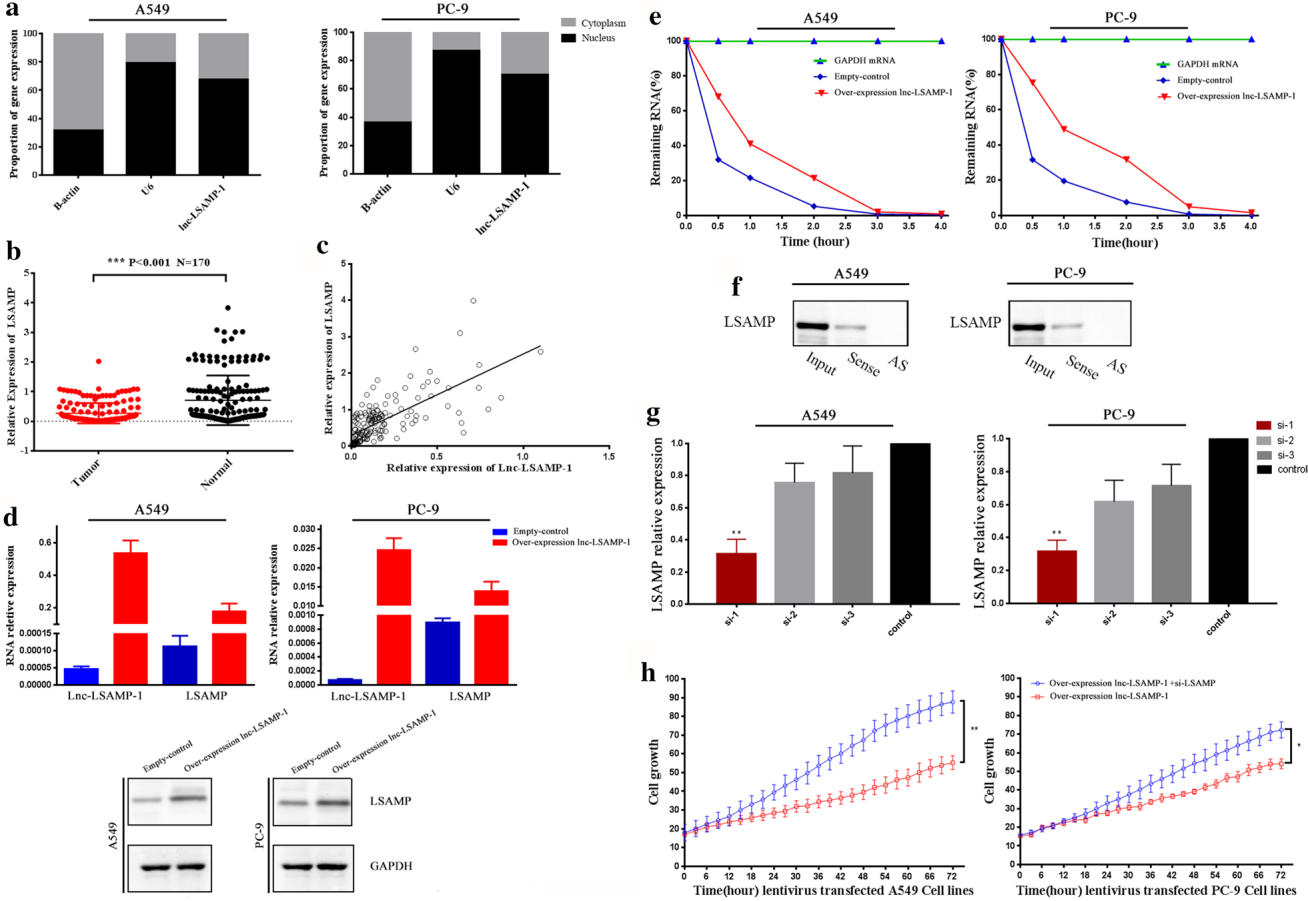
*Lnc-LSAMP-1* upregulates LSAMP by stabilizing LSAMP mRNA. **a** Subcellular localization of *Lnc-LSAMP-1* expression. **b** *LSAMP* gene expression levels in 86 pair-matched tumor tissues and adjacent normal tissues measured by qRT-PCR. **c** The correlation between *Lnc-LSAMP-1* and *LSAMP* gin 143 pairs of lung cancer tissues. **d** High expression of *Lnc-LSAMP-1* and *LSAMP* gene in A549 and PC-9 cells that transfected with overexpressed *Lnc-LSAMP-1*. **e** Over-expressed A549 and PC-9 cells and control were treated with actinomycin D (the concentration of actinomycin D was 2 mg/L). **f** The efficiency of si (*LSAMP* gene) measured by qRT-PCR. **g** The cell proliferation rate was compared with or without si-*LSAMP* in rescue assay. Significance was defined as p < 0.05 (*p < 0.05; **p < 0.01; ***p < 0.001)

Previous studies have verified that lncRNAs could exhibit cis-regulatory properties with their nearby coding-genes [33]. Interestingly, among thousands of potential targets, we noticed the limbic system-associated membrane protein (LSAMP) gene was significantly associated with Lnc-LSAMP-1 up-regulated (Fig. 5a). LSAMP is an important membrane protein and acts as a mediator in cell signaling. Emerging lines of evidence have shown that LSAMP serves as a tumor suppressor in multiple cancers [34–37]. Thus, we assumed that *Lnc-LSAMP-1* inhibited NSCLC growth and metastasis by up-regulating LSAMP gene. To confirm the cellular localization of *Lnc-LSAMP-1*, subcellular distribution assay was further performed. As the Fig. 6a shown, the expression of *Lnc-LSAMP-1* was determined to mainly concentrate in the cell nucleus, and a partial in the cytoplasmic fractions of A549 and PC-9 cells, respectively. To further explore the potential target genes for *Lnc-LSAMP-1*, we analyzed the correlation between *Lnc-LSAMP-1* and target genes from GEPIA web server. We found that the *LSAMP* gene is the most relevant gene (r = 0.78, *P* < 0.001, Additional file 2: Fig. S2a and S2b). According to GEPIA, *LSAMP* was down-expressed in NSCLC tissues compared with that of normal tissues (*P* < 0.05, Additional file 2: Fig. S2c). Furthermore, the levels of *LSAMP* gene were also down-regulated in lung tumor tissues than those in adjacent non-cancerous tissues (*P* < 0.001, Fig. 6b). Dramatically, the expression of *Lnc-LSAMP-1* was positively associated with *LSAMP* gene expression in NSCLC tissues (r = 0.699, *P* < 0.001, Fig. 6c). Besides, the cells with over-expressed *Lnc-LSAMP-1* showed distinctly higher levels of *LSAMP* expression while compared to control cells both at mRNA and protein levels (Fig. 6d). All the above results indicate that *Lnc-LSAMP-1* is positively correlated with the *LSAMP* gene.

Bioinformatics analysis indicated that *Lnc-LSAMP-1* is located in the downstream of the *LSAMP* gene and has partial sequences overlapping *LSAMP* transcripts (Additional file 2: Fig. S2d). So, we suspected that *Lnc-LSAMP-1* might affect *LSAMP* mRNA stability. Over-expressed *Lnc-LSAMP-1* cells and control cells were treated with actinomycin D (the concentration of actinomycin D was 2 mg/L). The results indicated that the degradation rate of *LSAMP* gene in cells with over-expressed *Lnc-LSAMP-1* was lower than that of the control groups (Fig. 6e). In addition, as shown in Fig. 6f, *Lnc-LSAMP-1* could directly bind to *LSAMP* (Fig. 6f*)*, which suggested that *Lnc-LSAMP-1* may have the function of interacting *LSAMP* gene to protecting its degradation.

### *The knock down of LSAMP gene promotes cell proliferation rate *in vitro

The efficiency of siRNAs targeting *LSAMP* gene were measured by RT-qPCR and the results showed that si-1 could achieve 70% of inhibition (Fig. 6g), so we selected si-1 for the following rescue experiments. The results demonstrated that the decrease in cell proliferation mediated by *Lnc-LSAMP-1* upregulation could be rescued by knocking down of *LSAMP* gene (*P* < 0.05, Fig. 6h).

### Lnc-LSAMP-1 enhances the susceptibility of TKI

**Fig. 7:**
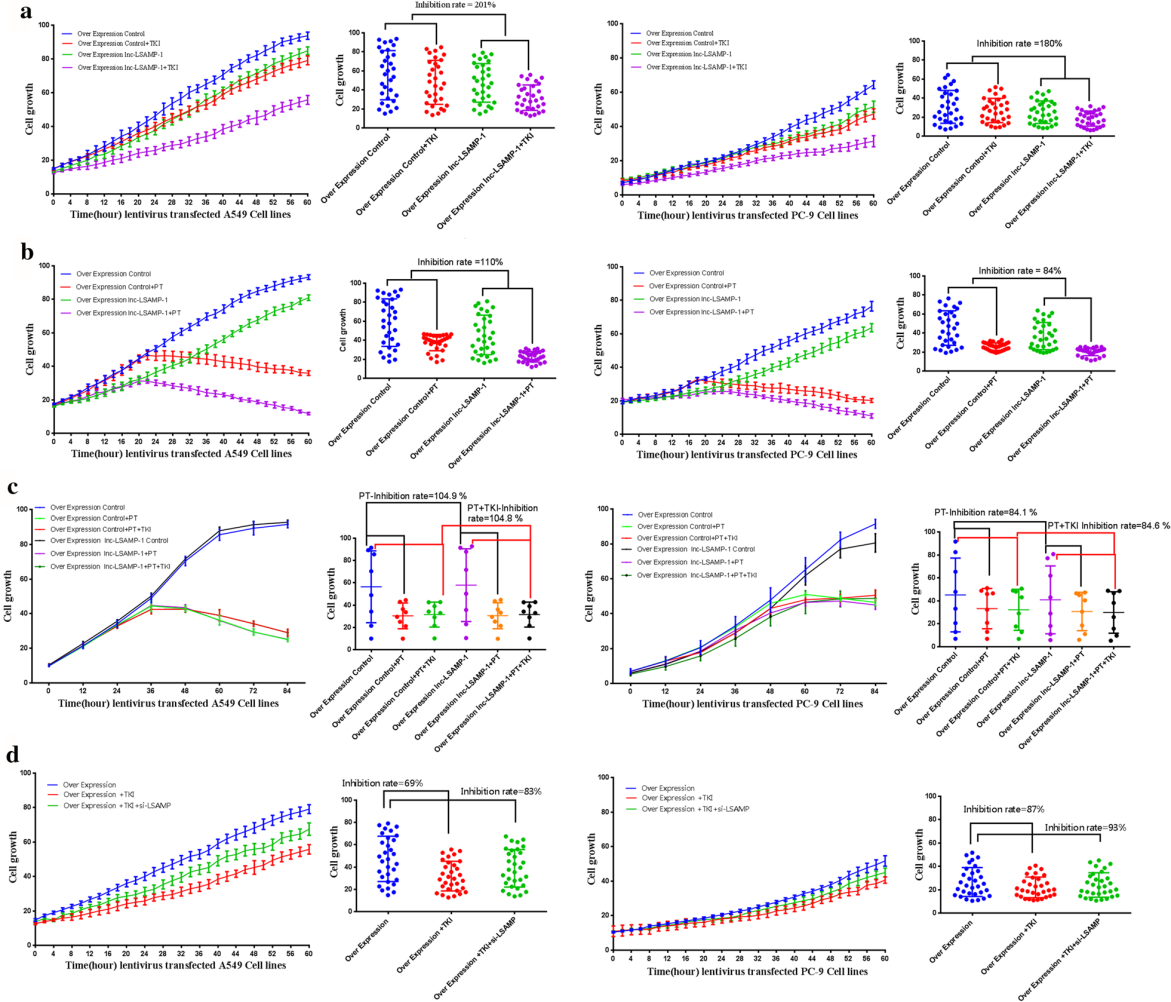
*Lnc-LSAMP-1 enhances the susceptibility of TKI treatment.* **a** The inhibition rate of TKI treatment in A549 and PC-9 cells. **b** The inhibition rate of cisplatin treatment in A549 and PC-9 cells. **c** The inhibition rate of TKI plus cisplatin treatment in A549 and PC-9 cells. **d** The inhibition rate of TKI treatment in A549 and PC-9 cells with silencing *LSAMP*

We further assessed the chemosensitivity effect of *lnc-LSAMP-1* expression on TKI or cisplatin treatment. Several TKI drugs including Tepotinib and Nilotinib were firstly used to comprehensively evaluate the effect of *lnc-LSAMP-1* on therapeutic sensitivity, and found that only Nilotinib but not Tepotinib (Additional file 3: Fig. S3) was observed to have fulfilling inhibition rate changes induced with *lnc-LSAMP-1* expression. The Nilotinib was then used for the subsequent assays. In the TKI treatment group, the cells with over-expressed *lnc-LSAMP-1* had a higher inhibition rate than that in empty control cells, with the ratio of 2.01 and 1.80 in A549 and PC-9 cells, respectively (Fig. 7a). However, no remarkable antibiotic susceptibility of *lnc-LSAMP-1* was observed in the cisplatin treatment group (Fig. 7b). Furthermore, the combined treatment of cisplatin and TKI was also investigated. Because of the cytotoxicity effect of cisplatin, the drug sensitivity induced by *lnc-LSAMP-1* did not dramatically observe both in A549 and PC-9 cells (Fig. 7c). Furthermore, the rescue experiments showed that silencing *LSAMP* expression could partially attenuate the cell cytotoxicity of *Lnc-LSAMP-1* under TKI treatment. All the results demonstrated that the suppression in cell proliferation mediated by *Lnc-LSAMP-1* upregulation could be rescued by knocking down of *LSAMP* gene (Fig. 7d).

## Discussion

A growing of evidence has substantiated that lncRNAs are extensively intricate in the tumorigenesis and cancer progression. Nevertheless, the detailed molecular mechanisms of lncRNAs in NSCLC remain unclearly documented [38]. In the current study, we demonstrated for the first time that *Lnc-LSAMP-1 had a significant association with* NSCLC development and progression. Our findings indicated that *Lnc-LSAMP-1* was markedly down-regulated in lung tumor tissues and cell lines. The *Lnc-LSAMP-1* expression was negatively associated with stage and N status. Additionally, *Lnc-LSAMP-1* was identified as an indicator for predicting poor prognosis in NSCLC patients. Function assays demonstrated that overexpression of *Lnc-LSAMP-1* inhibited proliferation, viability, invasion and migration ability, arrested cell cycle and facilitated apoptosis. Furthermore, upregulated *Lnc-LSAMP-1* enhanced TKI inhibition of lung cancer cell proliferation. Mechanistically, *Lnc-LSAMP-1* upregulated *LSAMP* by stabilizing *LSAMP* mRNA, thus playing a crucial role in NSCLC progression. Therefore, these results suggest that *Lnc-LSAMP-1* exerts anti-tumorigenesis in the progression of NSCLC and might be a potential predictor of prognosis in patients with NSCLC.

There is still an abundant amount of uncharted lncRNAs remaining to be elucidated in terms of their roles on cancer progression [39]. Previous studies have proved that lncRNAs can exert multitudinous molecular mechanisms to regulate gene activity and protein function based on their nucleoplasmic localization [40]. Evidence also suggest that there are many opportunities for lncRNA synthesis to negatively affect a neighboring protein-coding gene [41], through a variety of patterns including interfering with transcription, mRNA maturation and mRNA stability or translation [42]. Our Subcellular localization assay indicated that the expression of *Lnc-LSAMP-1* was existed both in the nucleus and cytoplasm. We further identified that there was an overlap among the protein-coding gene termed *LSAMP* which is located in front of *Lnc-LSAMP-1* and they shared partially genomic sequence. According to our results, the expression of *Lnc-LSAMP-1* in lung cancer tissues is highly related to the *LSAMP* expression level, supporting the modulating role in cis of *Lnc-LSAMP-1* to *LSAMP*.

*LSAMP* gene, mapping inside the 3q13 region, encoded a self-binding, antibody-like cell surface adhesion protein [30] which has been characterized to be associated with various phenotypes previously [43]. A growing body of evidence has illuminated that *LSAMP* was inactivated and loss of expression due to DNA methylation modifications and acted as a tumor suppressor gene in osteosarcoma [44, 45], acute myeloid leukemia [25], renal carcinoma [46] and ovarian carcinoma [47]. Tale Barøy, et al. also reported that re-expression of *LSAMP* inhibits the growth of osteosarcoma cells by indirectly upregulating one or more of the genes HES1, CTAG2 or KLF10 [34]. In addition, *LSAMP* is reported to be associated with poor cancer survival [48]. *LSAMP* is one of the four IgLONs that constitutes the immunoglobulin superfamily. The IgLONs as cell adhesion molecules, are positively involved in modification of cell–cell recognition [49]. Chen et al. found that *LSAMP* has been recognized as a translocation breakpoint-spanning gene in familiar clear cell renal cell carcinoma by reducing cancer cell proliferation [46]. In this study, we also found a decreased expression of *LSAMP* in lung cancer, which was in accordance with previous study [50]. Silencing *LSAMP* was shown to partially restrain the inhibiting effect of over-expressed *Lnc-LSAMP-1* on cell proliferation and sensitivity to TKIs treatment. Based on above information, *LSAMP* may function as a tumor suppressor gene in lung cancer progression, and it is reasonable to speculate that *Lnc-LSAMP-1* influences a variety of cellular biological behaviors by regulating *LSAMP* gene.

Chemoresistance is a major clinical obstacle for effective cancer chemotherapy, which could cause poor prognosis in those patients with NSCLC. A few studies have documented the activities of lncRNAs in chemotherapy response in many malignancies [51, 52]. Tracing effective biomarkers and illuminating the underlying mechanism of these molecules in chemoresistance would result in novel strategies to improve patient’s response to chemotherapeutics. In the current study, our data presented that the ectopic expression *Lnc-LSAMP-1* could enhances the susceptibility of TKI treatment in NSCLC patients. However, the exact mechanisms by which *Lnc-LSAMP-1* regulated the chemoresistance are not well-known and require further intensive investigation.

## Conclusions

In summary, we identified a novel lncRNA *Lnc-LSAMP-1* acting as a tumor suppressor in NSCLC, and the lower expression of *Lnc-LSAMP-1* was relevant with tumor progression and poor prognosis. *Lnc-LSAMP-1* interacted with neighbour gene *LSAMP* to prevents it from degradation and thus played fatal roles in NSCLC progression. Our findings provide a better understanding of the role of *Lnc-LSAMP-1* in NSCLC and a potential therapeutic target and prognostic predictor against this malignancy.

## Supplementary Information

**Additional file 1:** **Figure S1.** The associations between *lnc-LSAMP-1* and lung cancer development and progression by public data analysis. (a) *Lnc-LSAMP-1* was down regulated in LUAD and LUSC by GEPIA data. *Lnc-LSAMP-1* expression was prominently relevant with T status (b), N status (c), and stage (d). (e) Kaplan-Meier analysis of lung cancer patients between different *lnc-LSAMP-1 *expression.

**Additional file 2:** **Figure S2.** The prediction of *lnc-LSAMP-1* potent target genes. (a) Predictive analysis of *lnc-LSAMP-1* target genes in the GEPIA data. (b) The correlation between* LSAMP *gene and* Lnc-LSAMP-1* in lung cancer tissues by GEPIA data. (c) *LSAMP-1* was down regulated in LUAD and LUSC by GEPIA data. (d) The positions of *LSAMP* gene and *lnc-LSAMP-1* overlap partially in the UCSC database.

**Additional file 3:** **Figure S3.** The susceptibility of Tepotinib treatment on NSCLC cells with over-expressed Lnc-LSAMP-1. (a) The inhibition rate of Tepotinib treatment in A549 cells with high Lnc-LSAMP-1 expression. (b) The inhibition rate of Tepotinib treatment in PC-9 cells with high Lnc-LSAMP-1 expression.

**Additional file 4: Table S1.** Sequence information of primers used in RT-qPCR assay.

**Additional file 5: Table S2.** The demographics and clinical feathers of studied lung cancer patients

**Additional file 6:** **File S1.** The candidate lncRNAs nearby the fragile 3q13.31 chromosome region and their associations with lung cancer risk and prognosis analyzed by using GEPIA platform.

## Data Availability

The datasets used and/or analysed during the current study are available from the corresponding author on reasonable request.

## Abbreviations

LSAMP: Limbic system-associated membrane protein
lncRNA: Long non-coding RNA
NSCLC: Non-small cell lung cancer
GEPIA: Gene Expression Profiling Interactive Analysis
qRT-PCR: Real-time quantitative reverse transcription PCR
TKI: Tyrosine kinase inhibitors
