# Supplementary figures and images for: Long non-coding RNA LSAMP-1 is down-regulated in non-small cell lung cancer and predicts a poor prognosis
Source: Cancer Cell Int. 2022 May 6;22:181. doi: 10.1186/s12935-022-02592-0 (PMC9074231; doi:10.1186/s12935-022-02592-0)

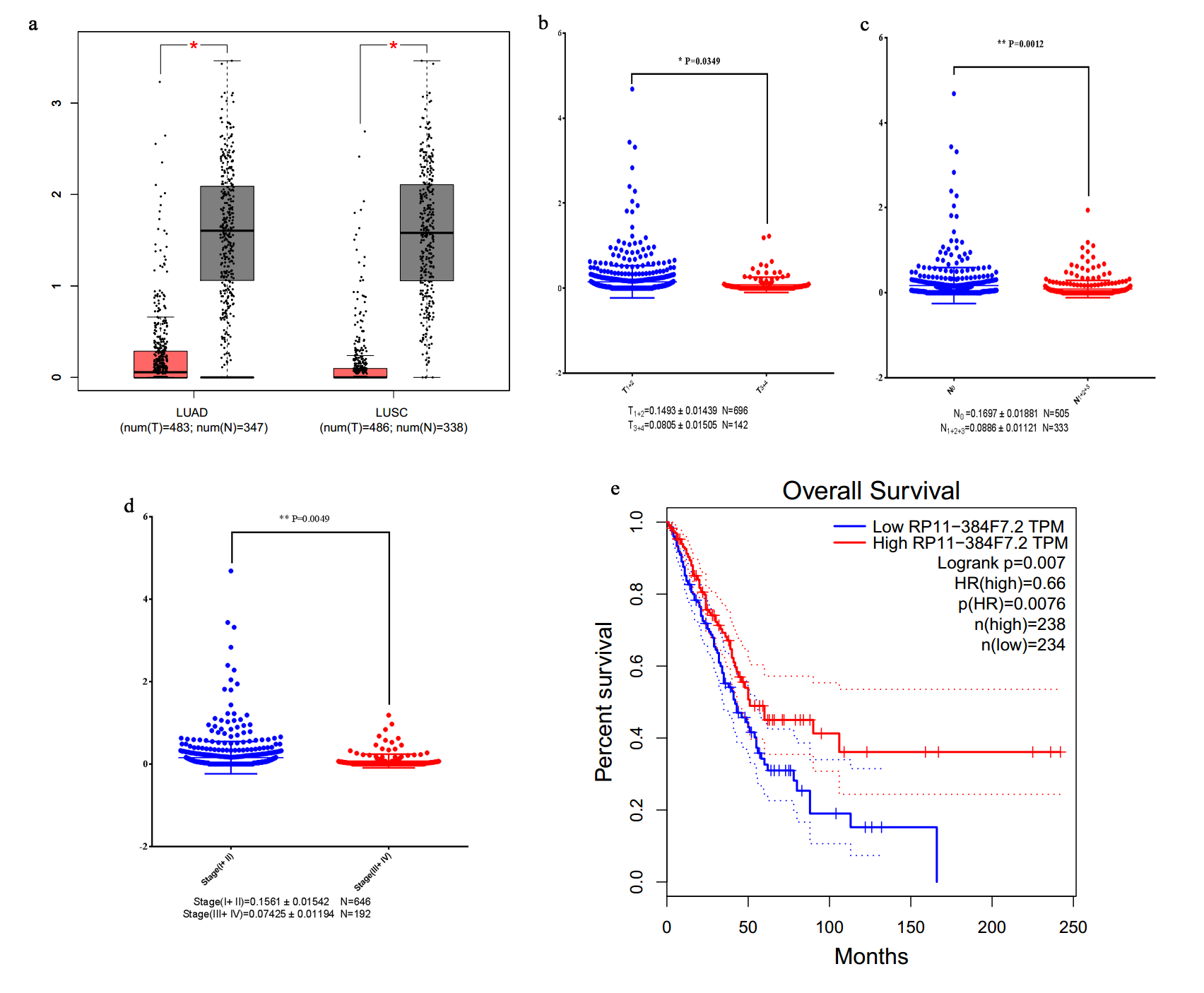

Supplement: Supplementary file 1 — Additional file 1: Figure S1. The associations between lnc-LSAMP-1 and lung cancer development and progression by public data analysis. (a) Lnc-LSAMP-1 was down regulated in LUAD and LUSC by GEPIA data. Lnc-LSAMP-1 expression was prominently relevant with T status (b), N status (c), and stage (d). (e) Kaplan-Meier analysis of lung cancer patients between different lnc-LSAMP-1 expression. [file 12935_2022_2592_MOESM1_ESM.tif]

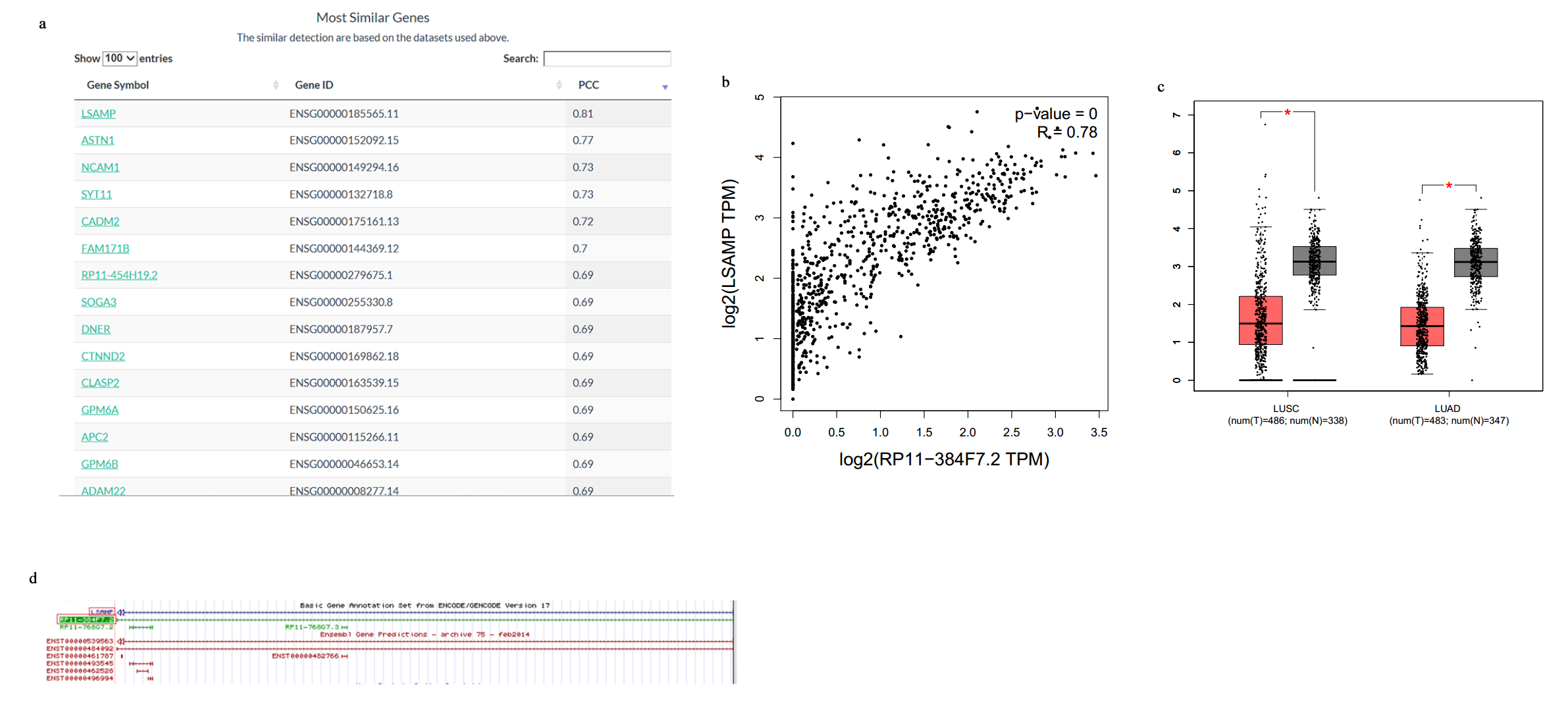

Supplement: Supplementary file 2 — Additional file 2: Figure S2. The prediction of lnc-LSAMP-1 potent target genes. (a) Predictive analysis of lnc-LSAMP-1 target genes in the GEPIA data. (b) The correlation between LSAMP gene and Lnc-LSAMP-1 in lung cancer tissues by GEPIA data. (c) LSAMP-1 was down regulated in LUAD and LUSC by GEPIA data. (d) The positions of LSAMP gene and lnc-LSAMP-1 overlap partially in the UCSC database. [file 12935_2022_2592_MOESM2_ESM.tif]

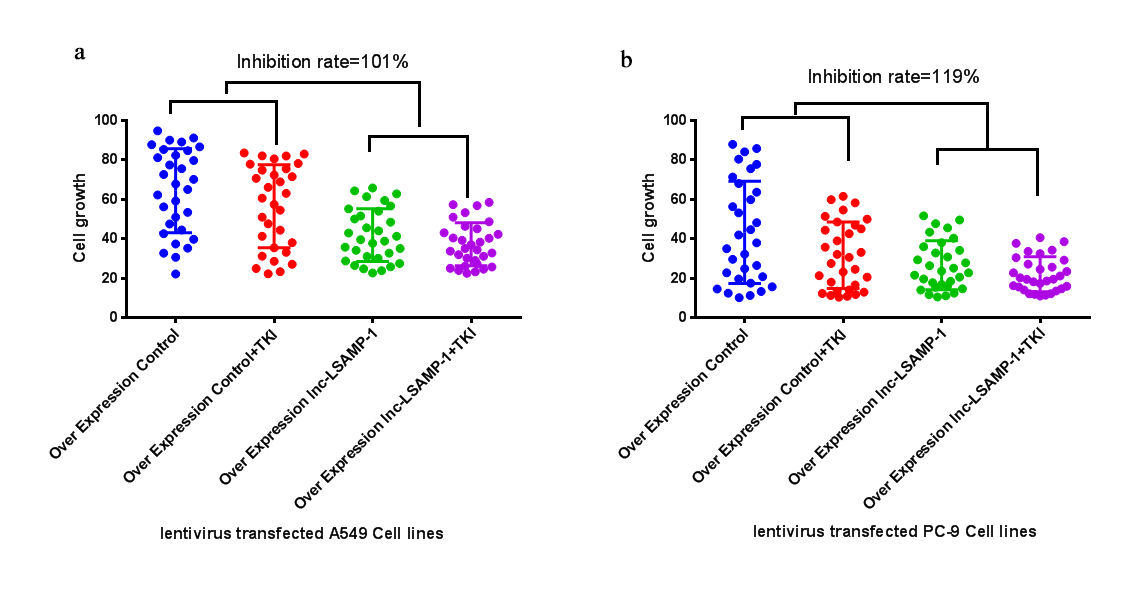

Supplement: Supplementary file 3 — Additional file 3: Figure S3. The susceptibility of Tepotinib treatment on NSCLC cells with over-expressed Lnc-LSAMP-1. (a) The inhibition rate of Tepotinib treatment in A549 cells with high Lnc-LSAMP-1 expression. (b) The inhibition rate of Tepotinib treatment in PC-9 cells with high Lnc-LSAMP-1 expression. [file 12935_2022_2592_MOESM3_ESM.tif]
