# Supplementary material for: Long non-coding RNA LSAMP-1 is down-regulated in non-small cell lung cancer and predicts a poor prognosis
Source: Cancer Cell Int. 2022 May 6;22:181. doi: 10.1186/s12935-022-02592-0 (PMC9074231; doi:10.1186/s12935-022-02592-0)
Supplement: Supplementary file 4 — Additional file 4: Table S1. Sequence information of primers used in RT-qPCR assay. [file 12935_2022_2592_MOESM4_ESM.doc]

**Table S1. Sequence of primers used in real time RT-PCR analysis.**

| **Gene Symbol** |  | **Forward primer** |  | **Reverse primer** |
| --- | --- | --- | --- | --- |
| *Lnc-LSAMP-1* |  | GTGTTTACACCAGGCACGGG |  | AACCCGGACAGACAAGCCTC |
| *LSAMP* |  | AGAGTTCAGCCGGATCGGAA |  | CGTGCCTCGGTTAAAATCCAC |
| β-actin |  | GGCGGCACCACCATGTACCCT |  | AGGGGCCGGACTCGTCATACT |
