# Supplementary material for: Long non-coding RNA LSAMP-1 is down-regulated in non-small cell lung cancer and predicts a poor prognosis
Source: Cancer Cell Int. 2022 May 6;22:181. doi: 10.1186/s12935-022-02592-0 (PMC9074231; doi:10.1186/s12935-022-02592-0)
Supplement: Supplementary file 5 — Additional file 5: Table S2. The demographics and clinical feathers of studied lung cancer patients [file 12935_2022_2592_MOESM5_ESM.docx]

**Table S2.** The demographics and clinical feathers of studied lung cancer patients

| Characteristic | Southern samples  N (%) | Eastern samples  N (%) | Pearson χ^2^ | | *P* value |
| --- | --- | --- | --- | --- | --- |
| Total | 115(67.6) | 55 (32.4) |  | | |
| Age | | | | | |
| <60 | 58 (50.4) | 30 (54.5) | 0.252 | | 0.616 |
| >= 60 | 57 (49.6) | 25 (45.5) |  |  |  |
| Gender | | | | | |
| Female | 31 (27.0) | 16 (29.1) | 0.085 | | 0.771 |
| Male | 84 (73.0) | 39 (70.9) |  |  |  |
| Family tumor history | | | | | |
| No | 102 (88.7) | 51(92.7) | 0.672 | | 0.412 |
| Yes | 13 (11.3) | 4 (7.3) |  |  |  |
| Smoking | | | | | |
| No | 39 (33.9) | 15 (27.3) | 0.757 | | 0.384 |
| Yes | 76 (66.1) | 40(72.7) |  |  |  |
| Stage(TNM) | | | | | |
| I+ II | 47(40.9) | 19(34.5) | 0.627 | | 0.429 |
| III+ IV | 68(59.1) | 36(65.5) |  |  |  |
| T status | | | | | |
| 1+2 | 71(61.7) | 24(43.6) | 2.825 | | 0.093 |
| 3+4 | 44(38.3) | 31(56.4) |  |  |  |
| N status | | | | | |
| 0 | 45(39.1) | 29(52.7) | 2.798 | | 0.094 |
| 1+2+3 | 70(60.9) | 26(47.3) |  |  |  |
| M status | | | | | |
| 0 | 83(72.2) | 37(67.3) | 0.430 | | 0.512 |
| 1 | 32(27.2) | 18(32.0) |  |  |  |
| Histological classification | | | | | |
| Adenocarcinoma | 60(52.2) | 22(40.0) | 2.418 | 0.299 | |
| Squamous carcinoma | 29(25.2) | 19(34.5) |  |  |  |
| Other types *^a^* | 26(22.6) | 14(25.5) |  |  |  |

*^a^* Large cell carcinoma, small cell carcinoma and hybrid or undifferentiated carcinoma.
